# Comparative phylogenomic insights of* KCS* and *ELO* gene families in *Brassica* species indicate their role in seed development and stress responsiveness

**DOI:** 10.1038/s41598-023-28665-2

**Published:** 2023-03-02

**Authors:** Uzair Muhammad Khan, Iqrar Ahmad Rana, Nabeel Shaheen, Qasim Raza, Hafiz Mamoon Rehman, Rizwana Maqbool, Iqrar Ahmad Khan, Rana Muhammad Atif

**Affiliations:** 1 Department of Plant Breeding and Genetics, University of Agriculture Faisalabad, Faisalabad, 38000 Pakistan; 2 Centre for Advanced Studies in Agriculture and Food Security, University of Agriculture Faisalabad, Faisalabad, 38000 Pakistan; 3 Center of Agricultural Biotechnology and Biochemistry, University of Agriculture Faisalabad, Faisalabad, 38000 Pakistan; 4 Precision Agriculture and Analytics Lab, National Centre in Big Data and Cloud Computing, Centre for Advanced Studies in Agriculture and Food Security, University of Agriculture Faisalabad, Faisalabad, 38000 Pakistan; 5 Institute of Horticultural Sciences, University of Agriculture Faisalabad, Faisalabad, 38000 Pakistan

**Keywords:** Molecular biology, Plant sciences

## Abstract

Very long-chain fatty acids (VLCFAs) possess more than twenty carbon atoms and are the major components of seed storage oil, wax, and lipids. *FAE* (*Fatty Acid Elongation*) like genes take part in the biosynthesis of VLCFAs, growth regulation, and stress responses, and are further comprised of *KCS* (*Ketoacyl-CoA synthase*) and *ELO* (*Elongation Defective Elongase*) sub-gene families. The comparative genome-wide analysis and mode of evolution of *KCS* and *ELO* gene families have not been investigated in tetraploid *Brassica carinata* and its diploid progenitors. In this study, 53 *KCS* genes were identified in *B. carinata* compared to 32 and 33 *KCS* genes in *B. nigra* and *B. oleracea* respectively, which suggests that polyploidization might has impacted the fatty acid elongation process during *Brassica* evolution. Polyploidization has also increased the number of *ELO* genes in *B. carinata* (17) over its progenitors *B. nigra* (7) and *B. oleracea* (6). Based on comparative phylogenetics, *KCS,* and *ELO* proteins can be classified into eight and four major groups, respectively. The approximate date of divergence for duplicated *KCS* and *ELO* genes varied from 0.03 to 3.20 million years ago (MYA). Gene structure analysis indicated that the maximum number of genes were intron-less and remained conserved during evolution. The neutral type of selection seemed to be predominant in both *KCS* and *ELO* genes evolution. String-based protein-protein interaction analysis suggested that bZIP53, a transcription factor might be involved in the activation of transcription of *ELO*/*KCS* genes. The presence of biotic and abiotic stress-related cis-regulatory elements in the promoter region suggests that both *KCS* and *ELO* genes might also play their role in stress tolerance. The expression analysis of both gene family members reflect their preferential seed-specific expression, especially during the mature embryo development stage. Furthermore, some *KCS* and *ELO* genes were found to be specifically expressed under heat stress, phosphorus starvation, and *Xanthomonas campestris* infection. The current study provides a basis to understand the evolution of both *KCS* and *ELO* genes in fatty acid elongation and their role in stress tolerance.

## Introduction

*Brassicaceae* is a vast family of plants including 372 genera and 4,006 species contributing to condiments, biofuel, food, oil, and fulfilling fodder demands for the ecosystem^1^. This group is also known for polyploidy studies due to diploid parents and allotetraploid species^2^. Over time U-triangle developed on polyploidization of *Brassica*’s proved its usefulness in studying the evolution of various genes and phenotypes^3^. The *B. carinata* (Ethiopian mustard, BBCC, 2n = 4X = 34) is an important species that contains 17 sets of chromosomes with a genome size of 1.087 Gb. It evolved after natural polyploidization between two diploid species *B. nigra* (BB, 2n = 2X = 16) and *B. oleracea* (CC, 2n = 2X = 18) about 0.047 MYA^4^. The *B. carinata* carries important agronomic and climate resilience characteristics including lodging, drought, and heat tolerance. Moreover, *B. carinata* has resistance against powdery mildew and white rust diseases highlighting its usefulness as a resistance resource in crop improvement^5^. Recently *B. carinata* has gained popularity as biofuel production in the United States and Canada^6^. However, its parents are valued for vegetables and condiments^7^. The *B. oleracea* being closest to *Arabidopsis thaliana* is considered an important *Brassica* species to study polyploidy^8^.

VLCFAs consist of 20 or more carbon atoms and are the key components of the cell membrane and cuticular lipids in plants^9^. Almost all plant cell types have VLCFAs and play a variety of crucial roles such as cell morphogenesis, energy storage in seeds, and stress responses^10,11^. Fatty acids of this group can be found abundantly in the form of suberins, sphingolipids, leaf cuticles, pollen epidermis, and cork cells. *Brassica* and Jojoba (*Simmondsia chinensis*) plants store them as a rich source of carbon^12^.

Biosynthesis of VLCFAs takes place in plastids and their elongation occurs in endoplasmic reticulum. During their biosynthesis, four key enzymes including ketoacyl-CoA synthase (KCS), 3-ketoacyl-CoA reductase, 3-hydroxyacyl-CoA dehydratase, and trans-2,3-enoyl CoA reductase performs elongation of fatty acid carbon chains^13–16^. There are four basic steps of chain elongation which includes (1) condensation, (2) l-reduction, (3) dehydration, and (4) ll-reduction. Condensation involves a reactant in the form of an acyl chain and in *Brassica*’s oleic acid reacts with malonate which is activated in the presence of coenzyme A by adding an extra carbon atom to the acyl chain and yielding β-ketoacyl. Later on, it passes through l-reduction, dehydration, and ll-reduction phases to convert activated β-ketoacyl to n + 2^17^. Two types of condensing enzymes are present in plants and they can be categorized into *KCS*-like and *ELO-*like gene families^12^. The basic mechanism of their interaction is still missing. However, comparative genomics and transcriptomics studies can help to understand their putative roles.

*KCS* gene family can be categorized into eight groups^11^. In *A. thaliana*, 21 *KCS* genes have been identified^18^ among which *KCS18* has been studied against the erucic acid biosynthesis which determines the edible oil quality^19^. Out of 21 KCS members, eight are characterized as *KCS-1, KCS2* (*DAISY*), *KCS-5* (*CER60*), *KCS-6* (*CER6*, *CUT1*), *KCS-10* (*FDH*), *KCS-13* (*HIC*), *KCS-18,* and *KCS-19 (FAE1)*^11^. Members of this family play vital roles in the biosynthesis of epidermal wax^20–22^, root suberin biopolymer^23^, seed stored triglycerides^19^ and maintain leaf guard cell density as well^24^. The *ELO* gene family was primarily identified in yeast (*Saccharomyces cerevisiae*). The *ELO* genes synthesize poly-unsaturated VLCFAs and were found only in lower plants^25–27^. In *A. thaliana,* only four *ELO* genes have been reported namely *At1g75000 (AtELO1), At3g06460 (AtELO2), At3g06470 (AtELO3),* and *At4g36830* (*AtELO4)*^28^. Until now, only *AtELO4* has been functionally characterized^29^.

Here, we have identified 118 *KCS* and 31 *ELO* genes in tetraploid *B. carinata* and its diploid progenitors *B. nigra* and *B. oleracea*. By employing various bioinformatics tools, we report their mode of duplication, phylogenetic reconstruction, genes structural organization, protein–protein interactions (PPI), promotor binding elements, and syntenic relationships during evolution. To broaden their perspective roles, we have also performed their expression profiling using various RNA-seq datasets comprising of heat stress, phosphate starvation, *X. campestris* inoculation, and during seed development and tissue specificity.

## Results

### Identification of *KCS *and *ELO* gene families in *B*.* carinata* and its progenitor species

The genomes of allotetraploid *B. carinata* and its diploid progenitors *B. nigra* and *B. oleracea* were searched against the queries of *B. napus* KCS and ELO proteins. Initially, 70 KCS proteins in *B. carinata*, 32 in *B. nigra*, and 33 in *B. oleracea* were identified. Later, these proteins were thoroughly scanned for the presence of conserved domains in both KCS and ELO proteins. Finally, 32, 33, and 54 un-truncated KCS proteins from *B. nigra*, *B. oleracea,* and *B. carinata* were retained, respectively. The same criteria opted for identification of ELO proteins and 17 of *B. carinata*, seven of *B. nigra,* and six of *B. oleracea* were found to be untruncated proteins. All these identified genes were renamed according to their chromosomal positions. A comparison of gene numbers among allotetraploid *B. carinata* and its diploid progenitors revealed that evolution has impacted both KCS and ELO proteins. Counting of gene numbers in *B. carinata* suggested that its genome might have lost 16 *KCS* and two *ELO* genes (Table S1).

Gene lengths of KCS ranged from 1.2 to 2.26 kilo base pairs (kbp) compared to ELO, which ranged from 0.57 to 4.39 kbp with amino acid lengths of 359–969 and 218–297, respectively. The maximum number of KCS and ELO proteins were found to be localized in endomembrane system and least number of proteins were predicted to be localized in nucleus and chloroplast (Table S2).

### Chromosomal distribution of *KCS *and *ELO* genes

**Figure 1:**
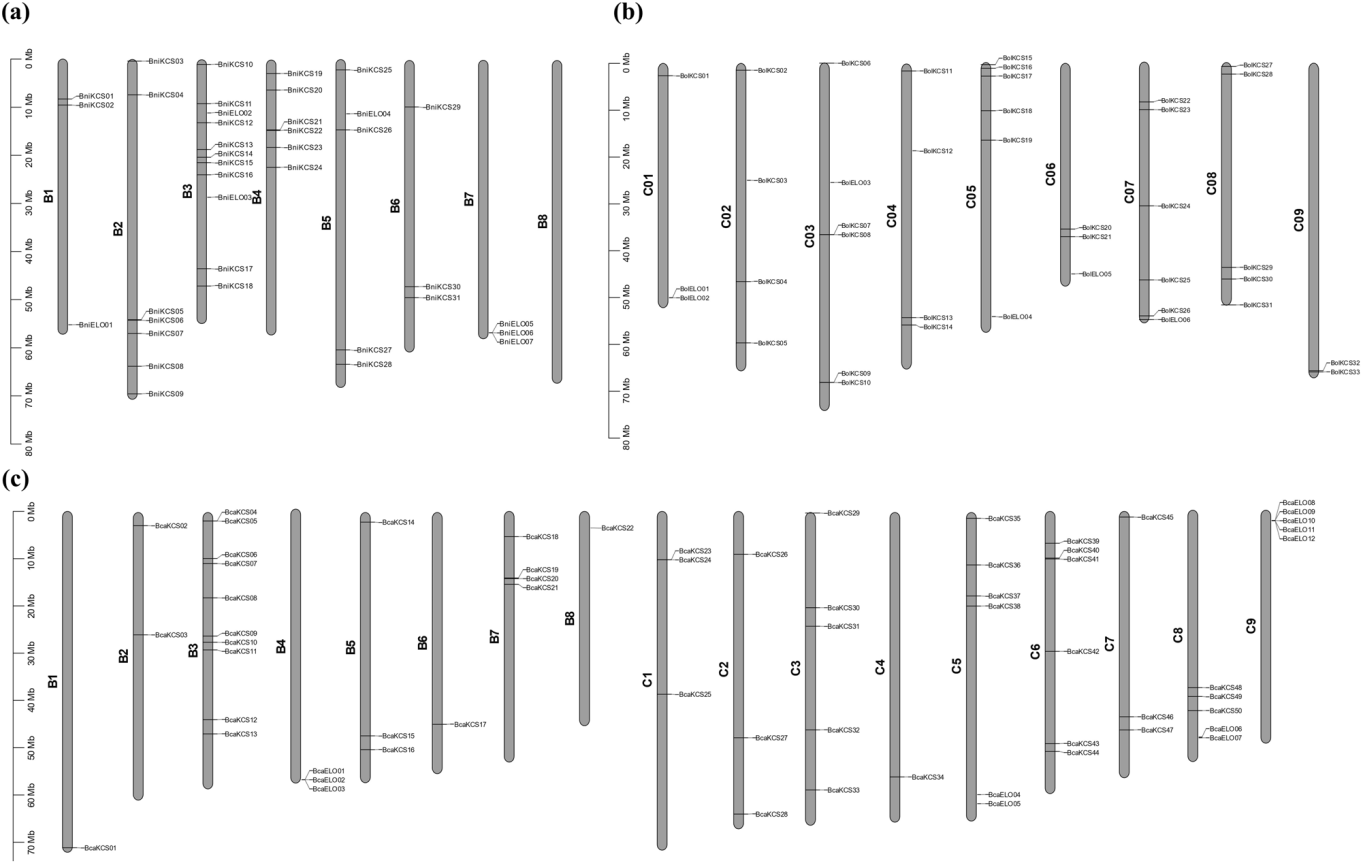
Localization of *KCS* and *ELO* genes on *Brassica* chromosomes; (**a**) *B. nigra* (**b**) *B. oleracea* and (**c**) *B. carinata*. The chromosomes are depicted by the grey bars, whereas labels on the bars indicate physical location of genes with reference to the scale.

In *B. nigra*, *KCS* genes were unevenly scattered on assembled chromosomes. Out of total eight chromosomes, first six (chrB01–chrB06) harbored 32 genes, whereas none of the genes were found to be localized on chrB07 and chrB08. The chrB03 contained maximum (9) genes, followed by chrB02 and chrB04 carrying seven and six genes, respectively (Fig. 1). In *B. oleracea* only six genes were clustered in paired form on chrC04 and chrC09 and the rest of the chromosomes harbored genes in random manner. The chrC05, chrC07, and chrC08 each contained five genes, whereas chrC01 carried only one gene.

In BB genome of *B. carinata*, chrB01, chrB06, and chrB08 each carried one *KCS* gene only and none of the *KCS* genes were mapped on chrB04 (Fig. 1). The chrB03 contained maximum (10) genes and this chromosome also carried maximum *KCS* genes in *B. nigra* genome. In the CC genome of *B. carinata*, chrC03 had maximum (5) genes, whereas chrC09 did not carry any *KCS* gene.

Interestingly, unlike the *KCS* genes, *ELO* genes were present in clusters indicating that these genes might be part of QTLs. Out of total 17 *BcaELO* genes, 12 were present in four clusters and the remaining five genes could not be localized on assembled chromosomes (Fig. 1). Moreover, in BB genome, *ELO* genes were mapped only on chrB04, whereas in CC genome these genes were localized on chrC05, chrC08, and chrC09. The *B. nigra* chromosomes had seven genes. The chromosomes chrB07 and chrB01 carried three and one genes, respectively, while other genes were identified from unsorted contigs, hence, could not be localized on chromosomes. Similarly, in *B. oleracea* genome, chrC03, chrC05, chrC06, and chrC07 each carried one *ELO* gene, whereas chrC01 contained two genes (Table S2).

### Intro-exon distribution of *KCS *and *ELO* genes

Gene structure was conserved throughout both gene families. Out of 118 *KCS* genes, 68 were intron-less, 32 genes were with a single intron, 13 were with 2 introns and 5 were with more than 2 introns, respectively (Fig. S1). Out of 32 *B. nigra* genes, 18 were intron less, whereas 11, and 2 genes had 1, and 2 introns, respectively. However, 9 introns were found in *BniKCS14* gene. In *B. oleracea BolKCS*20 gene had maximum 8 introns. Thirty-three genes were intron-less and 7, and 5 genes contained 1, and 2, introns respectively (Fig. S1). Similarly, in *B. carinata* 75 genes were intron less and 13 genes were with single intron, whereas 2 genes carried 2 introns, and the remaining 3 genes carried more than 2 introns. While maximum (7) introns were identified in *BcaKCS48* gene. Among three genomes the *B. nigra ELO* genes structure remained conserved during evolution. However, 4 genes of *B. oleracea* and 1 gene of *B. carinata* were identified to have introns (Fig. S2).

### Phylogenetic classification and motif analysis of KCS and ELO proteins in three *Brassica* spp.

**Figure 2:**
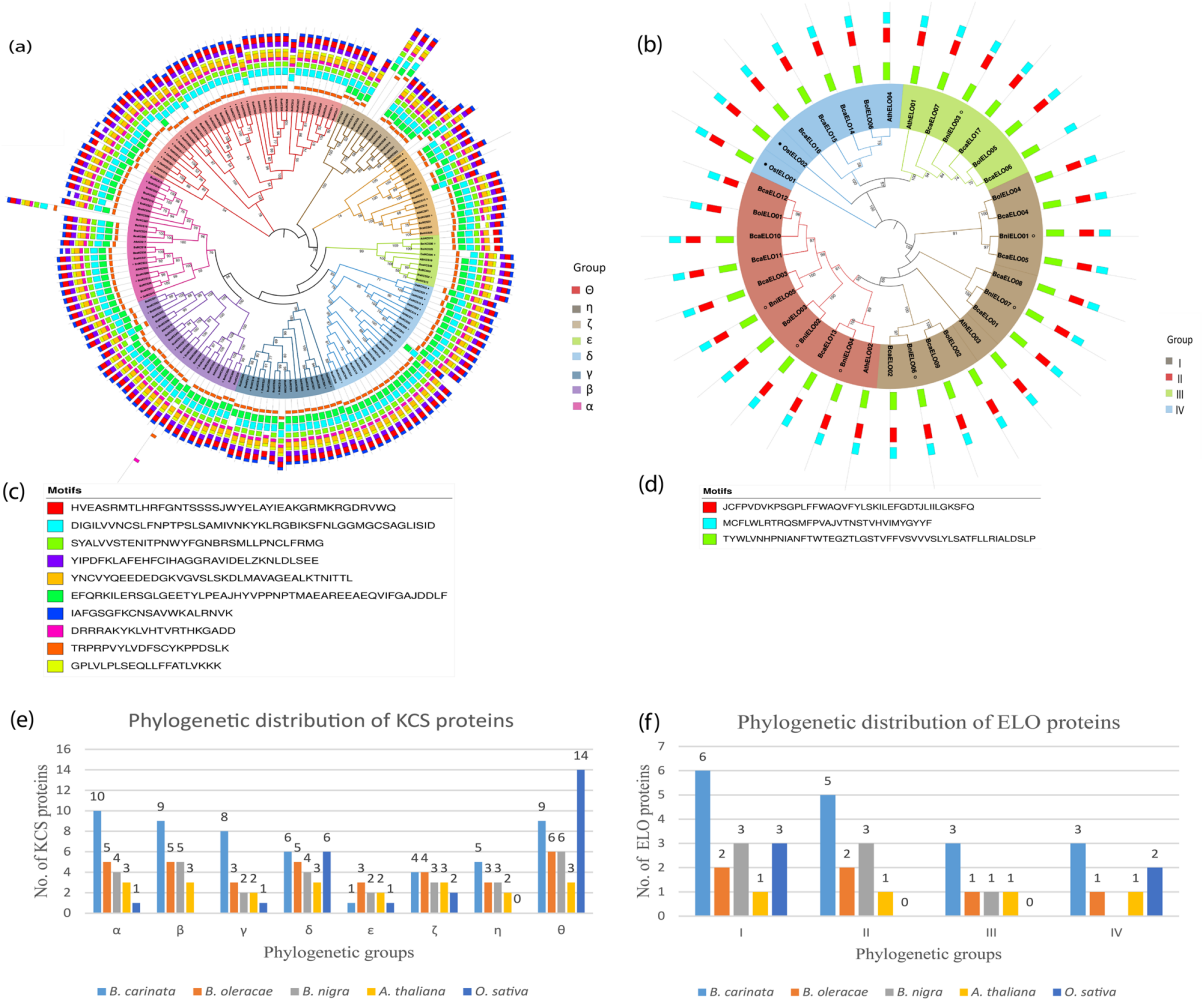
Phylogenetic classification and motifs distribution among KCS and ELO proteins in *B. carinata* and its progenitors. Phylogenetic grouping of (**a**) KCS-like genes and (**b**) ELO-like genes. Motifs identified among the (**c**) KCS and (**d**) ELO proteins, and group-wise distribution of (**e**) KCS and (**f**) ELO proteins in the *B. carinata*, its progenitors, *Oryza sativa* and *A. thaliana,* respectively.

Phylogenetic tree assists in determining the evolution and relationship of genes^30^. An unrooted comparative phylogenetic tree was constructed by using 50 KCS proteins from *B. carinata*, 32 from *B. nigra*, 33 from *B. oleracea*, 25 from *Oryza sativa,* and 21 from *A. thaliana*. Based on phylogenetic clades the KCS proteins grouped into eight groups (α, β, γ, δ, ε, ζ, η, and θ) (Fig. 2a). The θ clade contained maximum (38) members, followed by δ, β, α, γ, ζ, η, and ε which contained 24, 23, 23, 17, 16, 13, and 9 members, respectively (Fig. 2e). The α, β, and θ each had eight *KCS* orthologous gene pairs, whereas δ and η both contained 5 orthologous gene pairs. The two other remaining groups (ζ and ε) had two and one orthologous gene pairs, respectively. Moreover, constructing phylogeny also assists in assessing gene loss during evolution based on counting members in each clade. It could be inferred that 4 *KCS* genes from ε, 3 from ζ, and θ each, 2 from β and δ each and 1 from α and η groups each lost during evolution. However, members of γ group remained conserved during evolution.

Motif analysis was performed through MEME suit server to identify the characteristic regions of KCS and ELO proteins. The 10 and 3 types of motifs were identified in KCS (Fig. 2c) and ELO (Fig. 2d) protein sequences respectively. Interestingly, motifs orientation and numbers were highly conserved throughout the KCS proteins. However, genetic diversification among motifs was observed across members of all eight KCS clades. While seven KCS proteins; 4 from *B. oleracea*, 2 from *B. nigra,* and 1 from *B. carinata* exhibited variable motif structures as compared with other KCS proteins.

Same criteria for the construction of phylogenetic tree of ELO proteins was opted. Seventeen ELO protein sequences from *B. carinata*, 7 from *B. nigra*, 6 from *B. oleracea*, 2 from *O. sativa*, and 4 from *A. thaliana* were used for inferring comparative phylogenetic tree (Fig. 2b). The phylogenetic tree classified ELO proteins into four groups (I, II, III, and IV). Group I had 15 members, group II had 11 members, group III had 6, and group IV had 7 ELO protein members (Fig. 2f). Interestingly, group II and IV proteins were only present in *Brassica* species suggesting these might be *Brassica* specific groups. Moreover, absence of *O. sativa* members from these groups indicates that these groups evolved after separation of eudicots from monocots. Similar to KCS proteins, motif structures of ELO proteins were also conserved.

### Duplication analysis of *KCS *and *ELO* genes

Tandem and segmental types of duplications have their role in gene family’s evolution^31^. Gene pairs located on same chromosomes having a physical distance of < 50 kbp^32^ among them are said to be tandemly duplicated, while those pairs localized on different chromosomes are termed as segmentally duplicated^33^. A total of 6 *KCS* and 7 *ELO* paralogous gene pairs were found to be duplicated, of which 2 *KCS* and 4 *ELO* pairs were tandemly duplicated, whereas 4 *KCS* and 3 *ELO* gene pairs were found to be segmentally duplicated. The segmental duplications among the *KCS* genes were restricted to α and γ phylogenetic groups. Likewise, segmental duplications among *ELO* genes were also restricted to II and III phylogenetic groups (Table S1).

Moreover, nucleotide substitutions (Ka and Ks), duplication time, and selection type were also calculated among duplicated gene pairs. The Ka/Ks ratio of all duplicated pairs was < 1 suggesting that these pairs went through purifying selection to maintain their function. Moreover, all the duplicated pairs either tandemly or segmentally duplicated might have duplicated before the polyploidization of allotetraploid *B. carinata* as indicated from their calculated duplication time. Duplication time of tandemly duplicated pairs ranged from 1.14 to 18.19 million years ago (MYA), whereas duplication time of segmentally duplicated gene pairs was more recent ranging from 0 to 4.49 MYA (Table S1).

### Inter chromosomal syntenic relationships of *KCS *and *ELO* genes among *Brassicas* and *A. thaliana*

**Figure 3:**
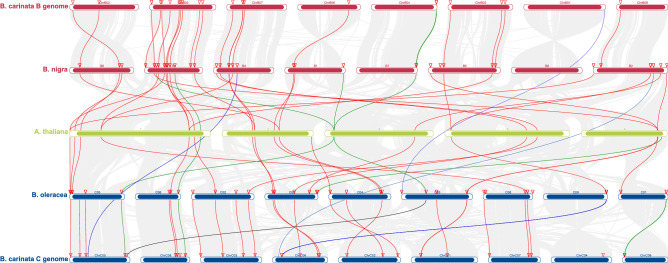
The *s*ynteny analysis among the AA, BB, and CC genomes of *Brassicaceae* family. The red bars are depicting BB chromosomes of *B. carinata,* and *B. nigra*, the green bars are of *A. thaliana* (AA), and the blue bars are for CC chromosomes of *B. carinata* and *B. oleracea*. The grey strings depict the collinear blocks, red and green lines are indicating orthologues identified in *KCS* and *ELO* genes and the blue and black lines are indicating possible translocations of *KCS* and *ELO* members.

The B2 chromosome of *B. nigra* and B8 of *B. carinata* were collinear. Chromosomal synteny analysis identified terminal deletions at both arms of *B. carinata* B2. The orthologs of five *B. nigra KCS* genes (*BniKCS03, BniKCS05*, *BniKCS06*, *BniKCS08*, and *BniKCS09*) might have been lost in *B. carinata* due to this deletion event. While another *BniKCS07* gene might be translocated, as its collinear gene was found on chromosome C6 of *B. carinata*. It could be hypothesized that this translocation might have happened before terminal deletion of B2 chromosome. The C7 of *B. oleracea* was collinear to C6 of *B. carinata* and the terminal end of short arm of C6 was showing collinearity with C7 of *B. carinata*. Interestingly non-collinear chromosomal region of C6 chromosome of *B. carinata* had three possible translocated *ELO* genes (*BcaELO08*, *BcaELO10*, and *BcaELO11*) from C9 of *B. oleracea*. The B5 chromosomes of *B. nigra* and *B. carinata* were collinear to each other and deletion in short arm of this chromosome might be responsible for removal of *BniKCS25* and *BcaELO13* genes, as their orthologs were identified from unassembled contigs in *B. carinata*. Similar types of possible translocations could also be observed in both *KCS* and *ELO* genes (Table S1 and Fig. 3).

### Prevalence of *Cis*-regulatory elements in promoters of *KCS *and *ELO* genes

*Cis*-regulatory elements are present in promotor regions and play their role in gene regulation^34^. Fifteen hundred base pairs upstream regions starting from start codon were used to identify *cis*-regulatory elements. In promoter regions of *KCS* genes, 23 types of elements were identified, and we classified them into four major classes: stress, developmental, light, and phytohormone-responsive elements (Table S3). The Myb elements were abundantly found in all *KCS* promoters and *BolKCS30* gene harbored maximum (12) Myb elements (Fig. S3). In *ELO* genes, 19 types of elements were identified and were classified into same four major groups as in *KCS* genes.

In *ELO* genes promoters, only two types of hormone-responsive elements (TGA-element, and DRE-core) were found. Developmental type of elements includes CAAT-box, CAT-box, and HD-zip 3 and playe their roles in endosperm and meristematic expression, and zein metabolism regulation, respectively^35^. The presence of a significant number of stress-responsive cis-regulatory elements in *KCS* and *ELO* promoters marks their involvement in stress responsiveness. MBS, STRE, as-1, DRE core, box s, W box, MYB recognition site, and MYB-like elements were common in both gene families and have roles in drought inducibility, heat stress, pathogenesis-related response, ABA- independent stress response, pathogenesis-related response, and anthocyanin production, respectively. The MYC elements were present in *KCS* genes only, whereas Myb and Myc were found in *ELO* genes only. Among phytohormone-responsive elements, only TGA and ABRE were common in both types of genes and were involved in auxin and abscisic acid responses, respectively (Fig. S4).

### Predicted protein secondary structures of KCS and ELO proteins

Secondary protein structures give insights into tertiary protein structure and also helps to understand protein functions and relationships^36^. Secondary proteins contained alpha helix, beta strands, TM helix, and disordered structures. The alpha helix % was dominant in both KCS and ELO proteins. All KCS proteins possessed more than 50% alpha helix, except for BolKCS11, BniKCS08, BolKCS25, and BolKCS44 which contained 39%, 47%, 47%, and 44% alpha-helix, respectively (Table S3). The percentage of beta-strands and TM helix % ranged from 9 to 15%, and 7 to 22%, respectively. Interestingly BolKCS32 protein did not carry a single TM helix. The range of disordered percentages was comparatively low and varied from 7 to 23% (Fig. S5).

Alpha helix was comparatively higher in ELO proteins and the lowest percentage of 69% was observed in BolELO03 protein. Like alpha helix, TM helix was also significantly higher and varied from 51 to 59% for ELO proteins (Fig. S6). However, the disordered structures and beta strands were comparatively lower in ELO proteins.

### Protein-protein interaction network analysis of KCS and ELO proteins

**Figure 4:**
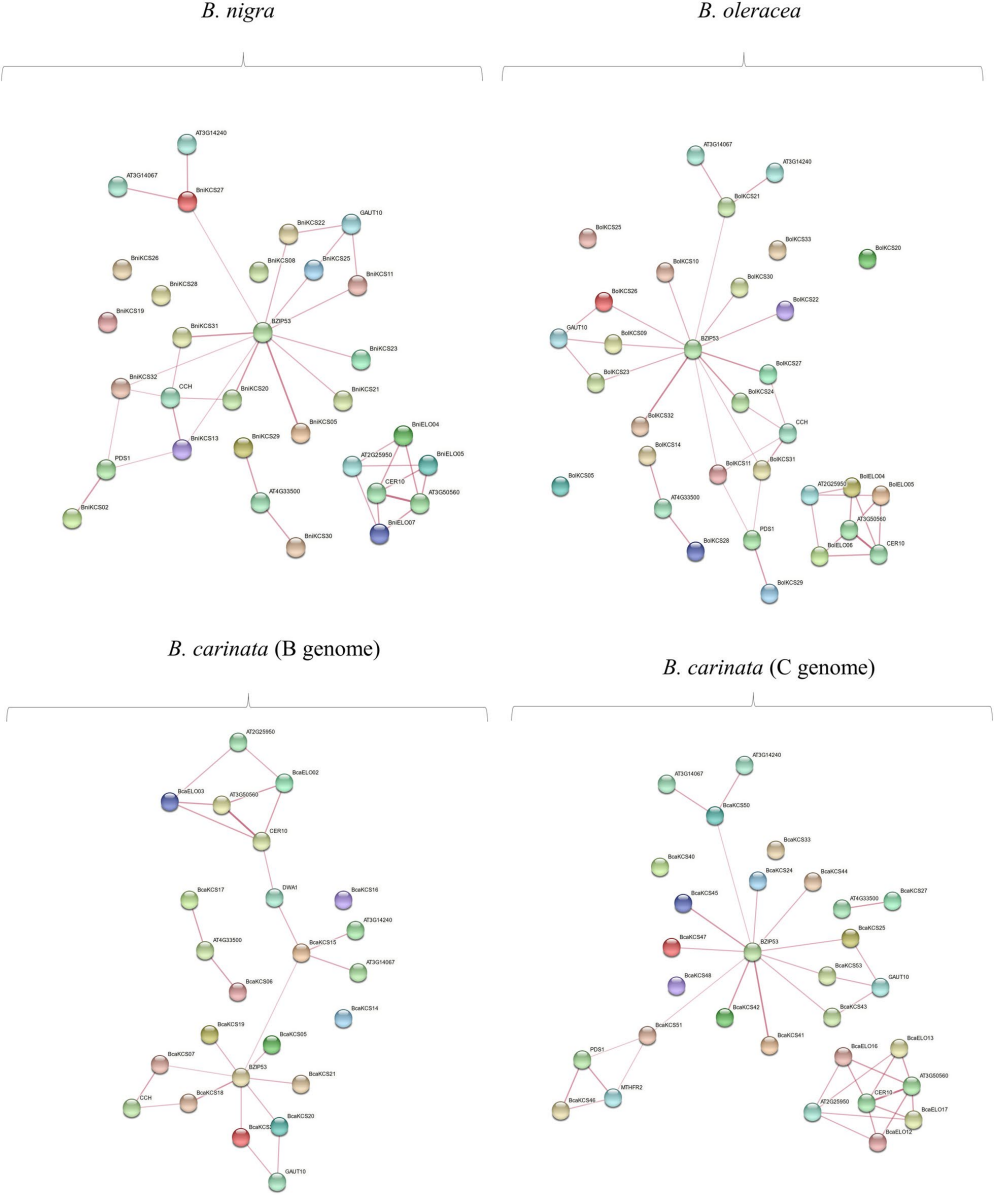
Predicted protein protein network of identified KCS and ELO proteins with other proteins. The strings and their intensity are indicating the interections of the members and the confidence estimate.

The PPI networks of KCS and ELO proteins were constructed through string database to know their potential roles and interactions. KCS proteins of *B. oleracea* interact with other four BolKCS proteins (BolKCS05, BolKCS20, BolKCS25, and BolKCS33) and showed no interaction with any other protein. Most of the KCS proteins showed a direct interaction with BZIP53 (Fig. 4), a transcription factor involved in regulation of seed maturation genes in *A. thaliana*^37^. Moreover, reports also showed that BZIP53 transcription factors have potential roles in biotic and/or abiotic stress responses^38–40^.

Furthermore, KCS proteins also interact with subtilase family proteins (AT3G14067, AT3G14240) galacturonosyltransferase (GAUT10), intracellular copper homeostasis (CCH), phosphatase 2C family proteins (AT4G33500), and PDS-1 (4-hydroxylphenylpyruvate). ELO proteins directly interact with NAD (P) binding rossmann-fold superfamily protein (AT3G50560), CER10, and AT2G25950 protein with unknown functions. In the tetraploid CC genome of *B. carinata,* KCS and ELO proteins showed the same interaction pattern. However, a new interaction of KCS protein with methylenetetrahydrofolate reductase protein was observed which is not found in PPI of *B. oleracea.* In BB genome of *B. carinata,* BcaKCS15 indirectly interacts with two ELO proteins; BcaELO03 and BcaELO02, which was not observed in other genomes. In *B. nigra,* both KCS and ELO proteins show the same interaction as in *B. carinata* and *B. oleracea* (Fig. 4)*.*

### Expression patterns of *KCS *and *ELO* genes in different tissues under normal conditions

**Figure 5:**
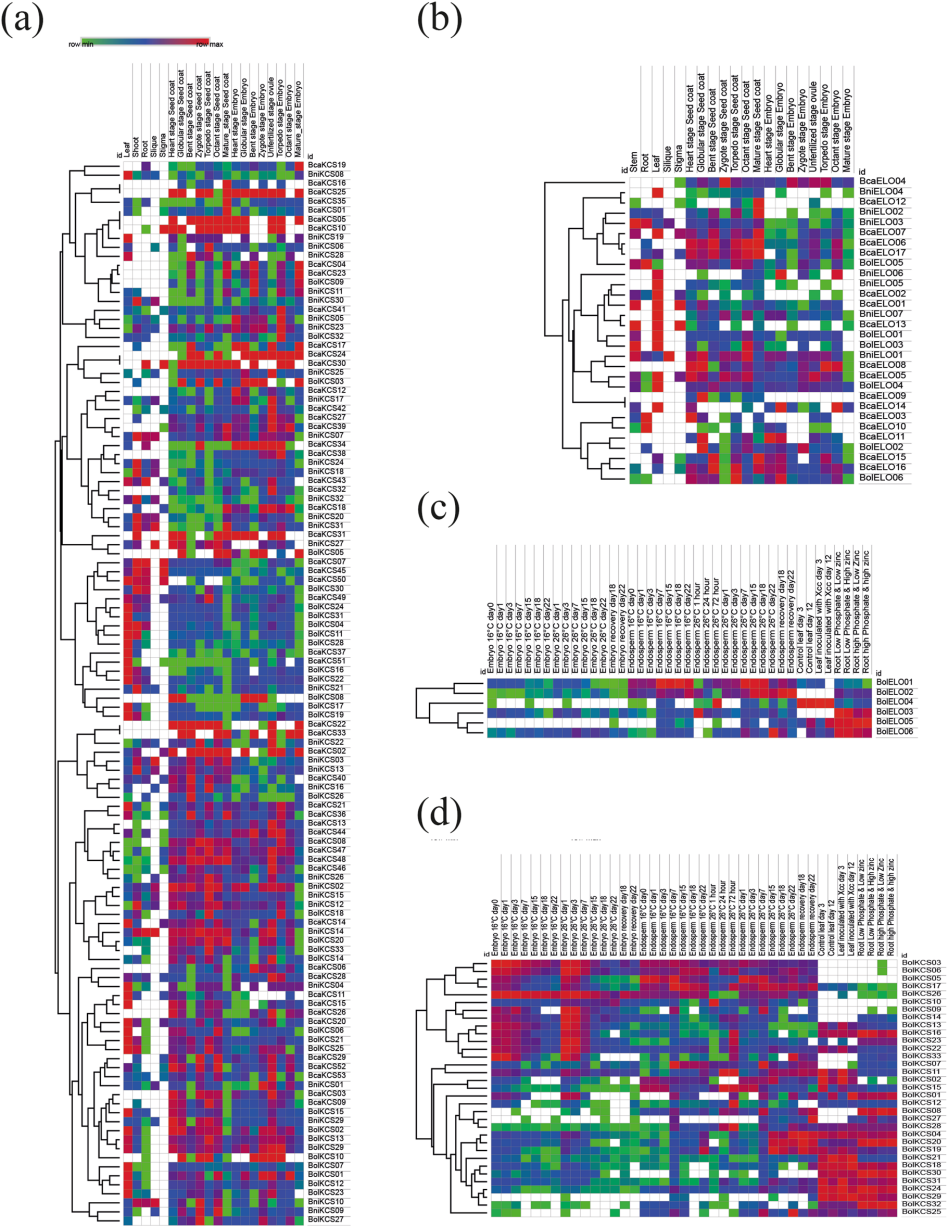
RNA-seq analysis based heat map of the identified *KCS* and *ELO* members: (**a**) *KCS* and (**b**) *ELO* genes expressions in different plant tissues of *B. carinata*, *B. nigra* and *B. oleracea*. The (**c**) *ELO* and (**d**) *KCS* genes expression levels under biotic and abiotic stresses in *B. oleracea*.

The expression patterns of *KCS* (Fig. 5a) and *ELO* (Fig. 5b) genes have been quantified in different tissues and during seed development stages. Among all three species, only eight *KCS* genes from *B. carinata* showed higher expression in shoot, root, and leaf tissues. During embryo development, three *KCS* genes (*BcaKCS24*, *BcaKCS25*, and *BcaKCS31*) showed an abundant expression throughout embryo development stages, whereas the expression of *BcaKCS34* and *BcaKCS44* decreased at mature seed stage. Most *KCS* genes exhibited that the transcription rate started decreasing when the embryo reached its maturity stage. Four *KCS* genes *BcaKCS04*, *BcaKCS23*, *BniKCS11*, and *BolKCS09* showed a unique expression rate at the mature embryo stage. However, *KCS* genes did not show expression in the seed coat. Three genes from *B. carinata*, (*BcaKCS02*, *BcaKCS22*, *BcaKCS33*), two genes from *B. nigra* (*BniKCS02*, *BniKCS14*), and only one gene from *B. oleracea* (*BolKCS02*) showed higher transcription rates during seed coat development. Comparatively *B. nigra* gene (*BniKCS02*) showed more transcripts than those of *B. carinata* genes. The transcription rate significantly decreased at mature seed coat stage. Reduction in transcription rate is obvious from *BniKCS02* and *BolKCS02* genes at mature seed coat. In silique tissues, only six genes from *B. nigra* (*BniKCS02*, *BniKCS03*, *BniKCS10*, *BniKCS14*, *BniKCS27*, and *BniKCS31)* got upregulated, whereas none of the genes from *B. carinata* and *B. oleracea* showed higher transcription rate. Upregulation of *B. nigra KCS* genes in silique tissues suggested that these genes have longer transcription cycle than other *KCS* genes (Table S4).

*ELO* genes showed different expression patterns in tissues as compared to *KCS* genes. None of the *ELO* genes from the three *Brassica* species were expressed at a higher rate in all tissues. Like *KCS* genes, expression of *ELO* genes also decreased in mature seed tissues. The lower expression of *ELO* genes during embryo development suggested their minor involvement in seed fatty acid biosynthesis. Two *ELO* genes (*BcaELO06* and *BcaELO17*) expressed at higher rate during seed coat development, whereas their expression was comparatively low during embryo development. Moreover, none of the *ELO* genes showed higher expression rate at mature embryo stage (Table S4).

### Expression patterns of *KCS *and *ELO* genes under biotic and abiotic stresses

Expression patterns under biotic and abiotic stresses have also been examined to know the response of *KCS* and *ELO* genes against different stresses. Among abiotic stresses, RNA seq data from embryo and endosperm tissues under different temperature regimes were acquired and analyzed. Data of embryonic tissues demonstrated that the majority of the *KCS* genes are thermo-sensitive, and their transcription rates also changed when tissues were exposed to different temperature regimes. Such patterns can also be observed in endosperm tissue under different temperature treatments. Overall, gene expression gradually decreased in embryonic tissues after 16 days, except at 16 °C where expression remained higher (Fig. 5c). Nine genes (*BolKCS03*, *BolKCS06*, *BolKCS09*, *BolKCS13*, *BolKCS14*, *BolKCS16*, *BolKCS17*, *BolKCS22,* and *BolKCS23*) showed higher expression at 16 °C after 0, 1, 3, 7, 15 and 18 days of embryo development (DAE). Similar upregulation of these genes was also observed at 22 °C after 1, 3, and 7 days of endosperm development. Upregulation of these genes suggested their roles in abiotic stress tolerance.

Eleven *KCS* genes (*BolKCS01*, *BolKCS04*, *BolKCS08*, *BolKCS18*, *BolKCS19*, *BolKCS20*, *BolKCS23*, *BolKCS24*, *BolKCS29*, *BolKCS30,* and *BolKCS32*) got upregulated in root tissues under 4 combinations of zinc and phosphate indicating their possible roles in zinc and phosphate uptake. Five genes (*BolKCS04*, *BolKCS24*, *BolKCS28*, *BolKCS29*, *BolKCS30*, *BolKCS31,* and *BolKCS32*) upregulated under *X. campestris* inoculation, zinc, and phosphate stresses, however, these genes did not show up-regulation in endosperm tissues and during seed development stages under heat stress treatments.

*ELO* genes had more diverse expression patterns than *KCS* genes. *BcaELO03*, *BcaELO05*, and *BcaELO06* were upregulated under zinc and phosphate stresses, whereas these genes were downregulated in embryo and endosperm tissues under heat stress treatments. Only one *ELO* gene (*BcaELO04*) was upregulated under *X. campestris* innoculation (Fig. 5d) (Table S5).

## Discussion

Due to paramount roles of fatty acid elongation-like genes in biosynthesis of VLCFAs, genome-wide identification of *KCS* genes has been performed in several plant species such as *A. thaliana*^11^ *Gossypium hirsutum* (cotton)^41^, *Hordeum Vulgare* (barley)^42^, *Arachis hypogaea* (peanut)^43^ and *Malus domestica* (apple)^44^. Although *KCS* genes have been reported in *B. napus*^45^ and its diploid progenitors, however, genome-wide identification of *KCS* genes in *Brassica* triangle is yet to be done. In this study, we identified *KCS* and *ELO* genes from whole genomes of *B. carinata* and its diploid progenitors *B. nigra* and *B. oleracea*. The *B. nigra*, *B. oleracea,* and *B. carinata* have 32, 33 and 53 *KCS* genes, respectively. The results highlighted that difference in number of identified *KCS* genes between diploid and tetraploid species might be due to terminal deletions in the tetraploid species chromosomes. Moreover, selection type estimates also highlighted that *KCS* gene went through purifying selections and lost their genomic parts related to KCS functioning. However, after polyploidization, *B. carinata* gained 4 *ELO* genes, as we identified a total of 17 ELO genes in tetraploid *B. carinata* genome, however, the sum of diploid progenitor’s *ELO* genes was 13^45,46^.

Phylogenetic analysis revealed eight sub-classes of KCS proteins including α, β, γ, δ, ε, ζ, η, and θ sub-classes. *Arabidopsis* KCS proteins can be seen in all these sub-classes^11^. Members of subclass δ shared sequence similarities with *AtKCS02* and *AtKCS20* which are reported to govern suberin biosynthesis^23^. Similarly, members of ε sub-class shared sequence homology with *AtKCS10* and *AtKCS15* which are involved in the growth and development of epidermal cells^10^. *Arabidopsis* proteins namely AtKCS05 and AtKCS06 are reported to be responsible for cuticular wax biosynthesis^22^ and *Brassica* proteins of related groups might also be involved in similar functions. The *AtKCS05* and *AtKCS06* genes shared high sequence similarity at protein level and their role in the elongation of carbon chain from C_24_ to C_28_ has been confirmed through heterologous expression in yeast and exhibited alike biochemical functions while transformation^47^. The β sub-class contained three *Arabidopsis* proteins; AthKCS08, AthKCS16, and AthKCS18 which have already been reported^11^. AthKCS16 causes elongation of carbon chain 34 (C_34_) to carbon chain 38 (C_38_), while AthKCS18 is the first KCS member to be characterized. The AthKCS18 induces the elongation of carbon chain from C20:1 to C22:1 and is predominantly expressed in seeds^48^, but in contrast to it, AthKCS16 was found to be mainly expressed in cauline leaves and rosette^49^ and proteins found in this group might be involved in elongation of VLCFAs. Gene structures of *ELO* and *KCS* genes varied from species to species.

Expression patterns of *KCS* and *ELO* genes were compared in different tissues under biotic and abiotic stresses. The *KCS* genes expressions were lower at mature seed stages as compared to earlier seed development stages. The *KCS1* gene in three *Brassica* species was expressed at higher rate in vegetative tissues^20^, while *KCS18/ FAE1* gene was mainly expressed in seed tissues and its expression decreased in mature embryos^48^. Moreover, *BnFAE1.1* and *BnFAE1.2* genes showed their transcripts in developing seed tissues only^50^.

In the current study, we have identified *KCS* and *ELO* sub-gene families in *B. carinata* and its progenitors. During polyploidization, tetraploid *B. carinata* lost its genomic part related to *KCS* genes, whereas *ELO* gene family expanded during this process. Gene structures showed that most of the genes were intron-less and *BniKCS30* was the longest gene with 22,659bp genomic sequence. RNA-seq analysis highlighted that expression of these seven genes including *BolKCS04*, *BolKCS24*, *BolKCS28*, *BolKCS29*, *BolKCS30*, *BolKCS31,* and *BolKCS32* were induced after *X. Campestris* inoculation and zinc & phosphate stresses. Functional characterization of these genes could provide novel resistant sources.

## Materials and methods

### Identification of *KCS and ELO* genes from *B. carinata, B. nigra, and B. oleracea*

Most updated genomic versions of *B. nigra* and *B. oleracea* were acquired from the Brassica database (BRAD) (brassicadb.cn) http://brassicadb.cn, while the most updated version of *B. carinata* was downloaded from (http://brassicadb.bio2db.com/). Full length proteins of *B. napus* i.e., BnaKCS (AOS88713.1), and BnaELO (XP_022565432) were acquired from the National Centre for Biotechnology Information (NCBI) database (https://www.ncbi.nlm.nih.gov) and used as query sequences to do BLASTp in TB tools (Toolbox for Biologist v 1.09832) with an E value < 1e^−5^ against whole peptides of three *Brassica* species^51^. All KCS and ELO proteins were identified by using BLASTp program of TB tools. Moreover, the conserved domain database (CDD) search was carried out to filter out the proteins-containing full-length conserved domains, whereas proteins with incomplete domains were discarded^52^. Subcellular localization of KCS and ELO proteins was predicted through Bologna Unified Subcellular Component Annotator (http://busca.biocomp.unibo.it).

### Gene structure and chromosomal localization of *KCS *and *ELO* genes

GFF file of each *Brassica* species was used to retrieve gene length, gene localization on the chromosomal strand, no. of coding sequences, and chromosome number in TB tools. Gene structures were predicted by putting the gene ids against the GFF file of each species. Moreover, molecular weight (kDa) and iso-electric point (*pI)* of identified proteins were retrieved from the Expasy compute *pI* tool available online(https://web.expasy.org/compute_pi/)^53^.

Relative gene localization on chromosomes was confirmed by the graphics program of TB tools^54^. The motif analysis was performed by MEME suit (https://meme-suite.org/meme/)^55^ and presented using iTOL (https://itol.embl.de/).

### Phylogenetics and gene divergence analysis

Identified proteins were multiple sequence aligned with Clustal omega^56^ and opened in MEGA X v 10.2.4^57^ to construct neighbor-joining phylogenetic tree with JTT (Jones-Taylor-Thornton) + G (Gamma distributed) model and 1000 bootstrap repeats^58^. A comparative tree consisting of three *Brassica* species, one non-*Brassicaceae* (*O. sativa*), and a model plant (*A. thaliana)* was constructed to explore the evolutionary relationship of KCS and ELO proteins.

Nucleotide substitutions causing amino acid changes are known to be non-synonymous (Ka) and those that do not cause changes are termed synonymous (Ks)^59^. The nature and magnitude of selection pressure occurring on coding sequences can be estimated through the ratio of Ka and Ks. The mode of selection and divergence time can also be determined through the Ks value^60,61^. A value of more than 1 (Ka/Ks > 1) indicates adaptive/positive selection^62^, less than one (Ka/Ks < 1) indicates negative selection^63^, whereas a value equal to one (Ka/Ks = 1) indicate neutral selection^64^.

### Prediction of secondary protein structure

The peptide sequences were uploaded to the Phyre2 online tool (http://www.sbg.bio.ic.ac.uk/phyre2/)^65^ and secondary structures were predicted based on homology.

### Promoter analysis of *KCS *and *ELO* genes

Fifteen hundred bp upstream regions from transcription start site were identified through TBtools by putting genomic FASTA and extracted sequences were submitted to the Plant Care database (http://bioinformatics.psb.ugent.be/webtools/plantcare/html) to identify the *cis*-regulatory elements^66^.

### Synteny analysis and protein-protein interaction

Whole-genome sequences and genome annotation files were put in the MCScanX tool^67^ to know the syntenic relationship between three *Brassica* species and model plant *A. thaliana*. The protein-protein interaction networks were predicted using string database (https://string-db.org/)^68^ by uploading the peptide sequences of three *Brassica* species.

### Global transcriptome profiling of *KCS *and *ELO* genes

To examine the expressions of *ELO* and *KCS* genes, tissue-specific RNA-seq data under normal and stressed conditions were retrieved from the European Nucleotide Archive database (https://www.ebi.ac.uk/ena/browser/home). Expression data of seed coat development stages (heart, globular, zygote, octant, bent, torpedo, and mature seed coat) and embryo development stages (heart, globular, zygote, octant, bent, torpedo, unfertilized stage ovule, and mature embryo) under normal conditions were acquired from Bio project PRJNA 641,876. Expression data of Bio project PRJNA 524,852 were used to examine gene expression during seed and embryo development under two different temperatures and day intervals. Root tissue data from Bio project PRJNA 524,852 under zinc and phosphate applications was also explored. Moreover, RNA seq data from Bio project PRJNA 421,190 was used to examine expression patterns under biotic stress of *X. campestris*.

The expressions of *KCS* and *ELO* genes in different tissues were quantified using galaxy Europe server (https://usegalaxy.eu/) and transcripts were evaluated in TPM (Transcripts per kilobase million)^69^. Moreover, heat maps were constructed using Morpheus software (https://software.broadinstitute.org/morpheus/).

## Supplementary Information

Supplementary Information.

## Data Availability

Genomes of *Brassica* species were acquired from (brassicadb.cn)http://brassicadb.cn, and (http://brassicadb.bio2db.com/) databases whereas queries sequences (BnaKCS (AOS88713.1), and BnaELO (XP_022565432) were retrieved from NCBI (https://www.ncbi.nlm.nih.gov/ ). RNA-seq data under normal (PRJNA641876,) and stressed conditions (PRJNA524852, PRJNA524852, PRJNA421190) were downloaded from ENA-EBI database (https://www.ebi.ac.uk/ena/browser/home).
