# Supplementary material for: Comparative phylogenomic insights of KCS and ELO gene families in Brassica species indicate their role in seed development and stress responsiveness
Source: Sci Rep. 2023 Mar 2;13:3577. doi: 10.1038/s41598-023-28665-2 (PMC9981734; doi:10.1038/s41598-023-28665-2)
Supplement: Supplementary file 1 — Supplementary Information. [file 41598_2023_28665_MOESM1_ESM.zip › Supplementary tables and files legend.docx]

**Figure S1:** Gene structure analysis of *KCS* genes of *Brassica* species. The phylogenetic tree with grouping and the green bars and gaps at its front is depicting the exons intron distribution.

**Figure S2:** Gene structure analysis of *ELO* genes of *Brassica* species. The phylogenetic tree with grouping and the green bars and gaps at its front is depicting the exons intron distribution.

**Figure S3:** The heat map showing the abundance of Cis regulatory elements of *KCS* genes*.*

**Figure S4:** The heat map showing the abundance of Cis regulatory elements of *ELO* genes*.*

**Figure S5:** Predicted secondary protein structure of KCS proteins.

**Figure S6:** Predicted secondary protein structure of ELO proteins.

**Table S1:** Evolution of identified *KCS* orthologous gene pairs in *B. carinata* and its progenitors.

**Table S2:** Molecular and physio-chemical properties of *KCS* and *ELO* genes in *B. carinata* and its progenitors*.*

**Table S3:** Cis- regulatory elements identified in *KCS* and *ELO* genes.

**Table S4:** Expression values (TPM) of KCS and ELO genes in different tissues.

**Table S5:** Expression values (log 2) of KCS and ELO genes in different tissues under biotic and abiotic stress conditions.
