# Supplementary figures and images for: Comparative phylogenomic insights of KCS and ELO gene families in Brassica species indicate their role in seed development and stress responsiveness
Source: Sci Rep. 2023 Mar 2;13:3577. doi: 10.1038/s41598-023-28665-2 (PMC9981734; doi:10.1038/s41598-023-28665-2)

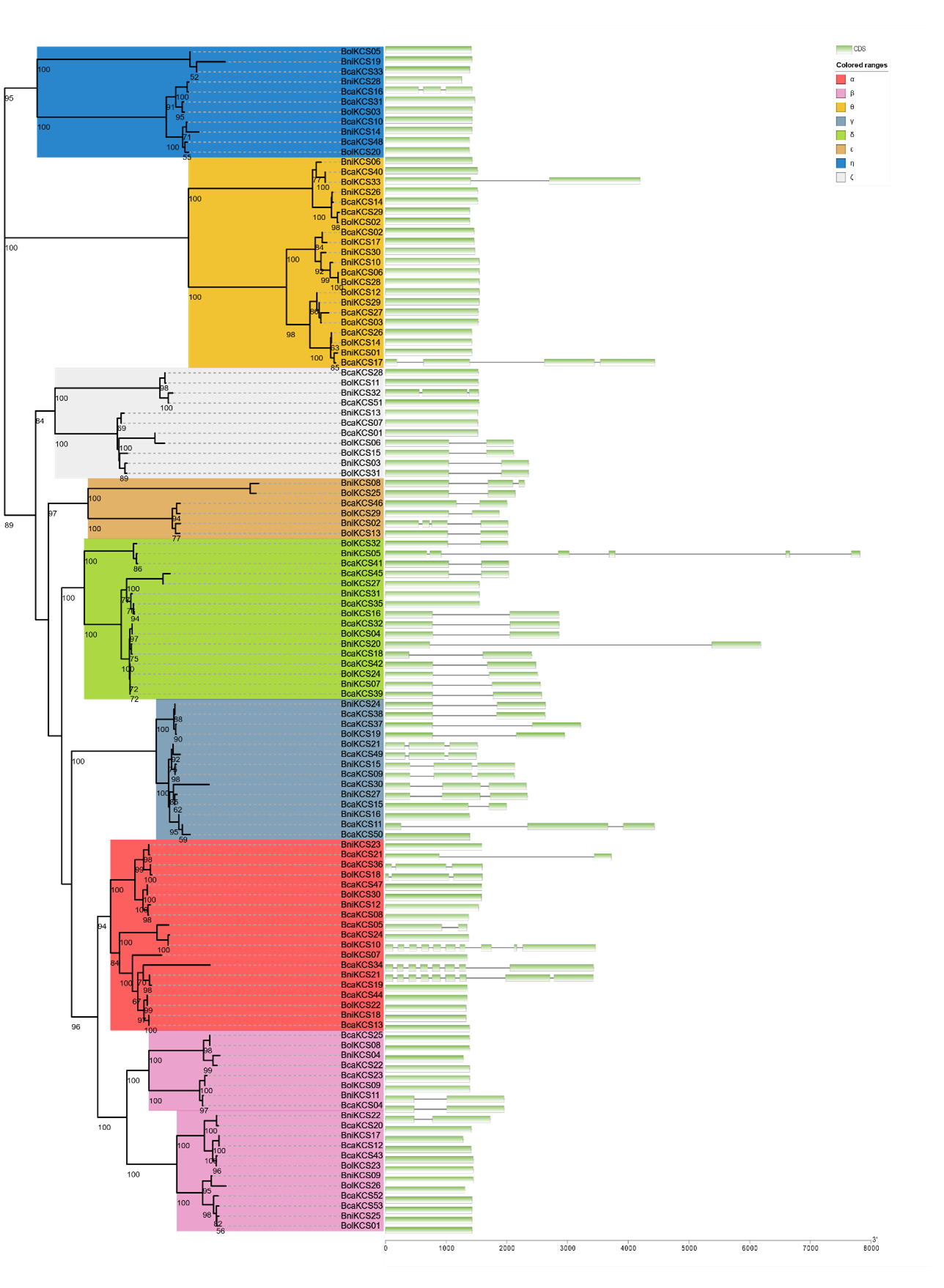

Supplement: Supplementary file 1 — Supplementary Information. [file 41598_2023_28665_MOESM1_ESM.zip › Figure S1.png]

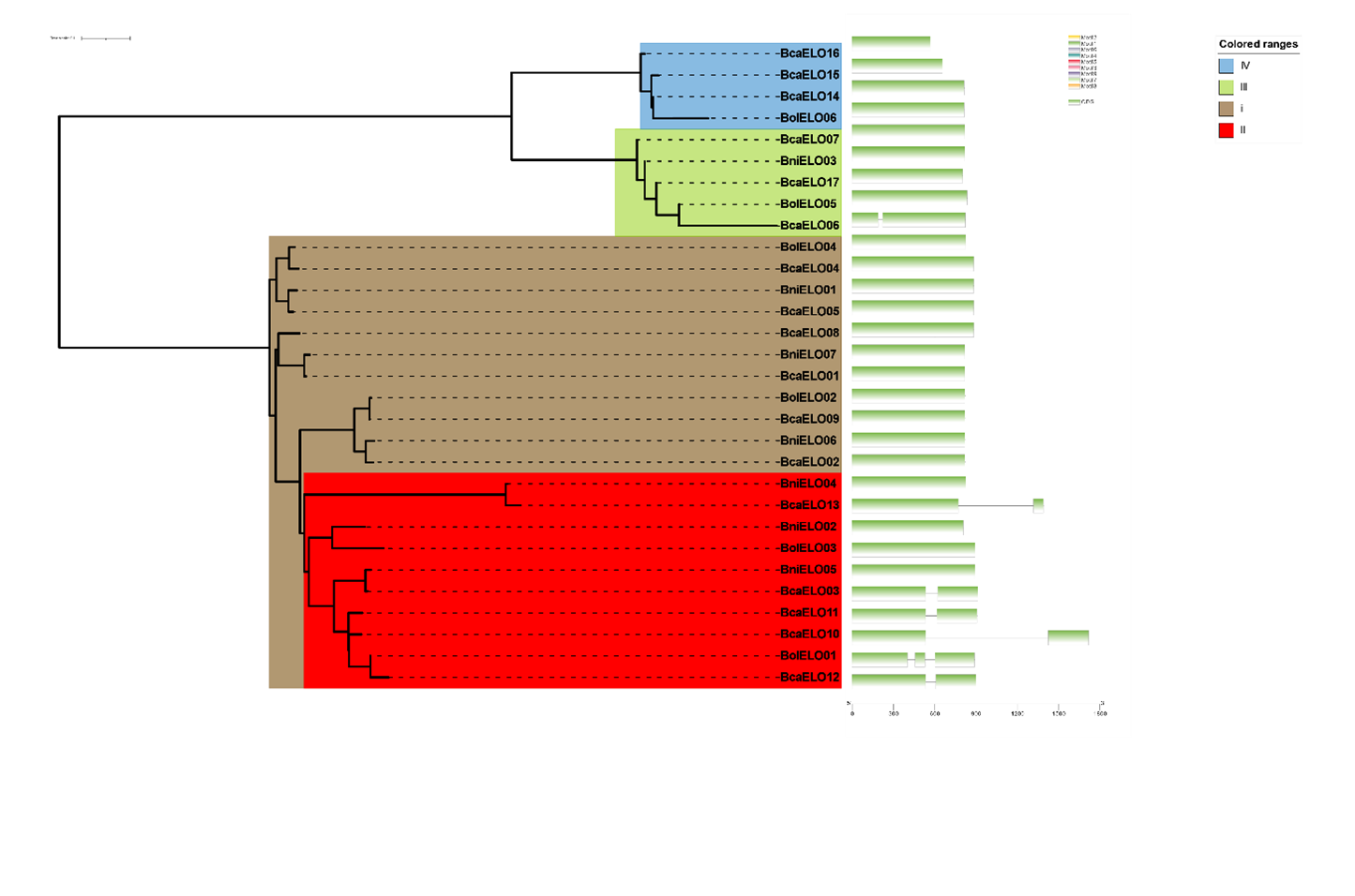

Supplement: Supplementary file 1 — Supplementary Information. [file 41598_2023_28665_MOESM1_ESM.zip › Figure S2.png]

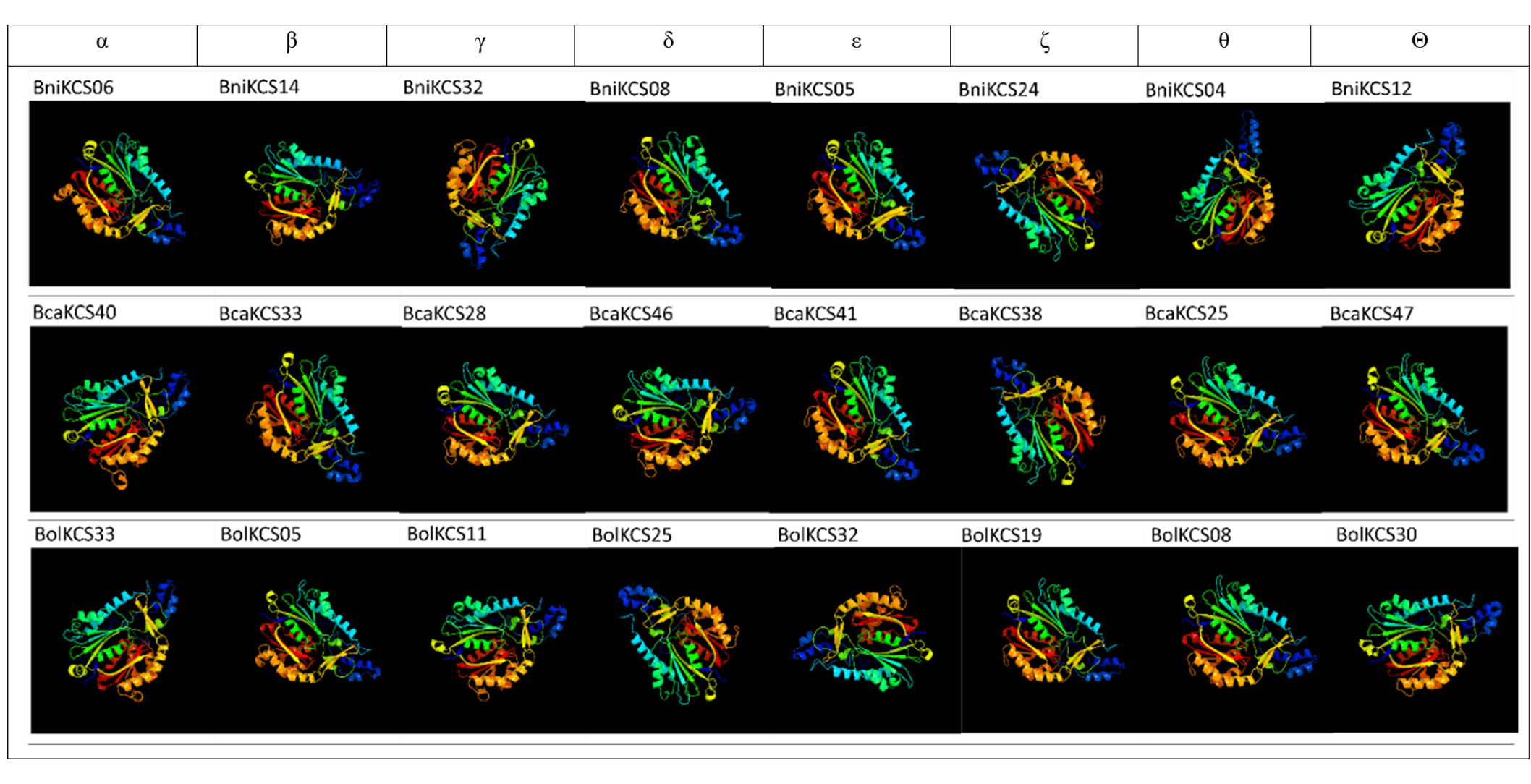

Supplement: Supplementary file 1 — Supplementary Information. [file 41598_2023_28665_MOESM1_ESM.zip › Figure S5.TIF]

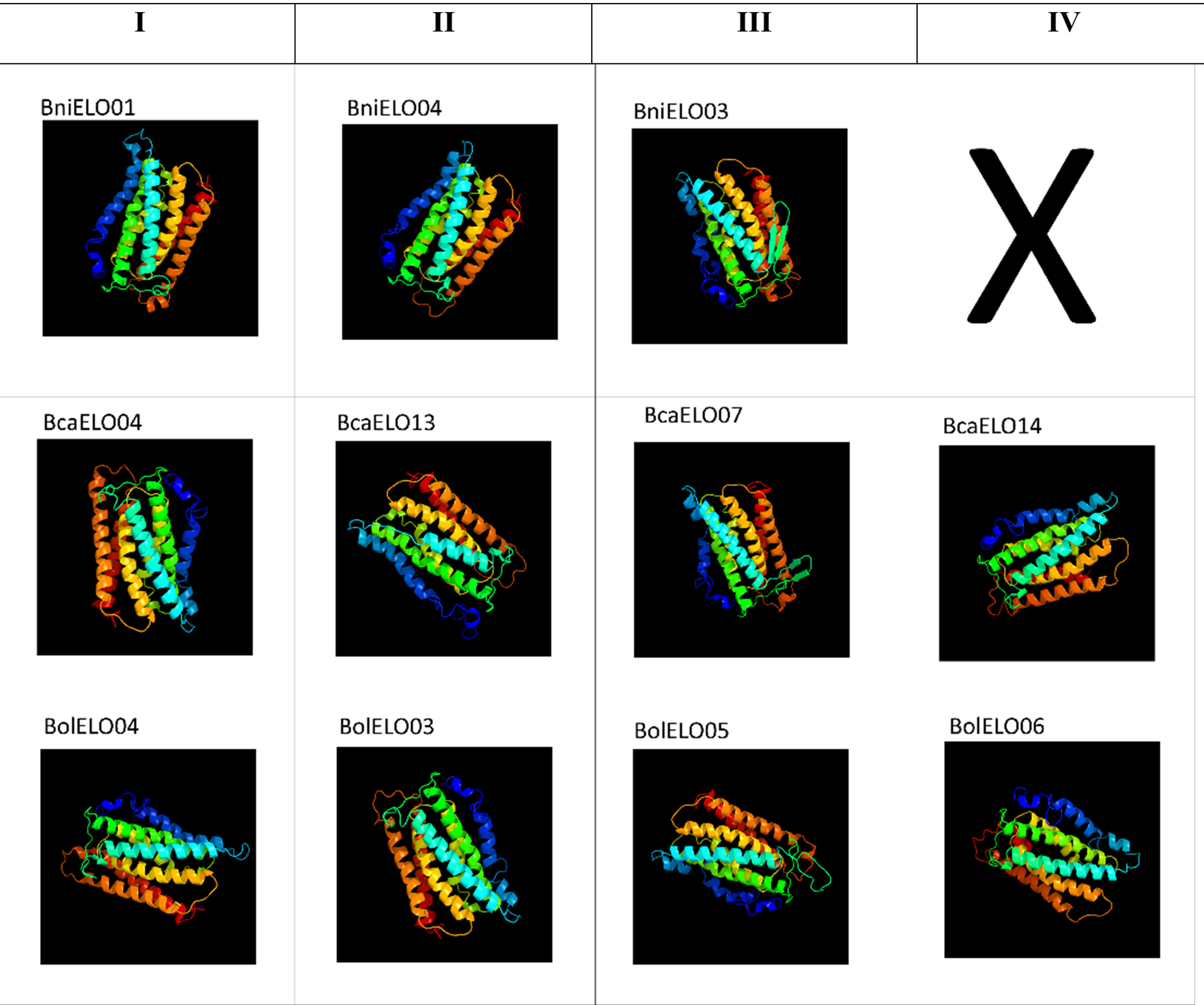

Supplement: Supplementary file 1 — Supplementary Information. [file 41598_2023_28665_MOESM1_ESM.zip › Figure S6.TIF]
